# Integrating Experimental Crystallization Kinetics into Autodesk Moldflow: Validation and Crystallinity Prediction for iPP and POM

**DOI:** 10.3390/polym18040482

**Published:** 2026-02-14

**Authors:** Vito Speranza, Valentina Volpe, Rita Salomone, Roberto Pantani

**Affiliations:** Department of Industrial Engineering, University of Salerno, Via Giovanni Paolo II, 132, I-84084 Fisciano, Italy; vavolpe@unisa.it (V.V.); rsalomone@unisa.it (R.S.); rpantani@unisa.it (R.P.)

**Keywords:** moldflow crystallization kinetics, iPP crystallization kinetics, POM crystallization kinetics, moldflow isothermal and non-isothermal simulation, moldflow crystallization analysis

## Abstract

An accurate prediction of the final properties of injection-molded semi-crystalline parts requires models that capture crystallization kinetics during processing. This work presents two practical strategies to incorporate experimentally derived crystallization behaviors into Autodesk Moldflow, addressing cases where kinetics differ from the software’s native Avrami–Hoffman–Lauritzen formulation. We apply these methods to isotactic polypropylene (iPP T30G) displaying heterogeneous nucleation with a low-temperature plateau, and to polyoxymethylene (POM) exhibiting combined heterogeneous and homogeneous nucleation. The parameters for Moldflow were obtained by matching isothermal half-crystallization times (t_0.5_) and by tuning flow-induced nucleation terms. Validation against isothermal and non-isothermal injection tests shows agreement between calculated and expected crystallinity evolution and reproduces measured spherulite diameters.

## 1. Introduction

One of the primary demands in the market of molded plastic products is obtaining higher performing components at reasonable costs. To achieve these goals, the reduction in the time needed to pass from design to production is undoubtedly a key point. In this view, injection molding numerical simulation is a powerful tool. Numerical simulation allows us to overcome the trial-and-error procedure that was largely used to determine the materials and conditions required to realize molded components with desired final properties [1]. Reduced simulation times, coupled with the possibility of having a more complete description of the material behaviors inside the simulations, allow us to accurately predict the variables’ evolution during the process, that in turn determines the final properties of the molded components [2,3,4,5]. Thus, faster and reliable simulations replace the time-consuming injection molding tests required by the trial-and-error procedure, reducing both the time to market and, thus, the costs.

A deeper description of the material behavior is achieved by increasing the number of considered phenomena that take place in the material and considering their interplay; this implies an increment in the number of material models adopted in the simulation software. For molded parts made using semi-crystalline thermoplastic materials, predictions of final properties improve when accounting for the crystallization phenomena. A crystallization analysis (which involves a model definition and its parameters identification) should be integrated into the simulation software to improve the accuracy of final properties predictions such as shrinkage and warpage [2,3,6].

For several decades, both the quiescent crystallization phenomenon [7,8,9] and the crystallization development during the process have been considerably investigated [10,11,12]. However, the adopted crystallization descriptions varied according to the different polymeric materials considered [13,14,15,16,17]. This difference can be ascribed to two main reasons. Firstly, the crystallization phenomenon is intimately connected to the nature of the material, and its chemical and physical properties determine the way (namely if, when, and how) it crystallizes. Secondly, the phenomenon was described following different approaches.

On the other hand, the final properties of the molded parts are dependent on the crystallinity and morphology developed during the process [18,19,20]. Therefore, for a long time now, open-source, commercial [3,21] and academic injection molding simulation software [2,22] has adopted models that can predict the evolution of the crystallinity and morphology in the semi-crystalline molded parts during the injection molding process. In such a way, reliable predictions of mechanical properties, shrinkage, warpage and optical properties of semi-crystalline molded part can be achieved.

In advanced simulation software, the kinetics model adopted to describe the crystallinity evolution is improved by taking into account the flow-induced crystallization phenomenon [2,6]. The Avrami–Kolmogoroff model is widely adopted by the simulation codes to describe the crystallinity evolution. The model considers crystallization as the combination of two processes—nucleation and growth. For the crystalline functions, namely nucleation density and growth rate, Hoffman–Lauritzen equations are typically adopted. However, to allow an accurate and material-specific prediction of crystallinity evolution during the injection molding process, model parameters have to be properly defined at least in quiescent conditions and preferably also under flow conditions [12,23].

In the commercial simulation codes, the parameters of the crystallization kinetics model are stored in the semi-crystalline materials database. Thus, to be able to describe the crystallinity evolution with commercial software, the polymer crystallization database must be generated by characterizing the experimental evolution of crystallinity in the material (at least under quiescent conditions) using the same exact kinetics model adopted by the simulation software. However, this approach presents at least two main drawbacks. Firstly, the crystallization kinetics model implemented in the simulation code may not be able to accurately describe the experimental crystallization behavior of the material. Secondly, identification of the model parameters for the simulation code database when a kinetics model already exists, but it differs from the crystallization kinetics model implemented in the simulation code.

In this work, we present two potential approaches to describe the crystallization behaviors of commercial semicrystalline polymers in the simplest manner using Moldflow. These behaviors include heterogeneous nucleation, characterized by a saturation value for the number of nuclei, and homogeneous nucleation overcoming the heterogeneous nuclei. While these two situations are not explicitly codified in Moldflow, they can be effectively described through straightforward and indirect methods. The present work aims to suggest strategies to integrate experimentally derived crystallization kinetics into Autodesk Moldflow when the material behavior cannot be directly described by the crystallization model implemented in the software. Two representative semicrystalline polymers are considered: an isotactic polypropylene characterized by heterogeneous nucleation with saturation at high undercooling, and a polyoxymethylene exhibiting a combined heterogeneous–homogeneous nucleation mechanism. For both materials, crystallization parameters for Moldflow are identified by matching experimentally determined isothermal half-crystallization times and by appropriately tuning the nucleation terms available in the software. The proposed approaches are validated through isothermal and non-isothermal simulations of injection molding, as well as by comparison with experimental crystallinity evolution and morphological data. The results demonstrate that, despite the intrinsic limitations of the Moldflow crystallization framework, accurate predictions of crystallinity development and spherulitic morphology can be achieved using simplified and transferable procedures, thus enhancing the reliability of process simulations for semicrystalline polymers.

## 2. Materials and Methods

Isotactic polypropylene (iPP) T30G, supplied by Basell (Ferrara, Italy), and Delrin 111PF Polyoxymethylene (POM), supplied by DuPont (Wilmington, DE, USA), characterized in previous works [24,25] were adopted. The commercial grades were used as received from the suppliers.

The injection molding (IM) tests for iPP were performed with HAAKE Minijet II by Thermo Scientific (HAAKE, Milan, Italy) using a rectangular cavity with the following dimensions: 1.0 mm thickness, 10.0 mm width and 60 mm length. The tests were performed with 200 °C injection temperature, an injection pressure P_in_ = 200 bar and it was kept constant for 5 s, while a non-constant mold wall temperature was used. Thin slices from the samples (100 µm thickness) were cut along the flow thickness plane by Leica RM 2265 slit microtome (Wetzlar, Germany) and observed by optical microscopy Olympus BX51 (Segrate, Milano, Italy) in polarized light. Sample slices were oriented at 45° with respect to the analyzer. Dimensions of spherulitic structures were evaluated analyzing optical micrographs with an image processing software (ImageJ 1.54p, Ashburn, VA, USA).

Experimental data, obtained by calorimetric and rheo-optical analysis under quiescent conditions, were used to develop the quiescent crystallization kinetics model of POM.

Injection molding simulations of both materials (iPP and POM) were performed with Moldflow software (Moldflow Insight Ultimate 2018, Autodesk Inc., San Rafael, CA, USA). To this aim, two different Moldflow database materials were created according to the characterization of the iPP and POM adopted in this work. In Moldflow simulations, the dual domain mesh was also selected to perform the crystallization analysis.

To determine the values of some parameters contained in the crystallization kinetics equations, a tuning was carried out by performing a series of isothermal simulation tests. Considering a constant and homogeneous temperature, changing one single parameter allowed us to determine the effect of that parameter on the crystallization kinetics.

## 3. Moldflow Crystallization Analysis

In order to improve the predictions of shrinkage and warpage, crystallization analysis has been considered in Moldflow software. Such an analysis is available only for products Insight Premium and Insight Ultimate when the solid model of the thin-walled part is described adopting the Dual Domain or the Midplane meshing technique. Instead, for thick parts with complex geometry, that must be described by adopting a 3D meshing technique, this simulation capability is not available. Predictions of relative crystallinity evolutions and final relative crystallinity distribution, or final average crystal size, are produced by Moldflow simulations of the thermoplastics injection molding process using a semi-crystalline material with appropriate crystallization morphology properties data. The crystallization model implemented considers the phenomenon characterized by two processes: nucleation and growth. The flow effect on the crystallization process is accounted for according to [26]. Furthermore, the interplay between crystallinity and other material properties (f.i. viscosity, density) is considered. In the following, a description of the crystallization model adopted by the Moldflow software is given.

Previous experimental investigation on iPP [27] observed that shear flow strongly affects the nucleation process and vice versa exhibits a relatively small effect on the structures’ growth. Thus, following Hoffman–Lauritzen theory [28,29], the growth rate depends only on temperature, T and pressure, P, according to

(1) $$G\left(T\right)=G_{0}\cdot exp\left[-\frac{U^{*}}{R_{g}\left(T-T_{\infty }\right)}\right]\cdot exp\left[-\frac{fK_{g}}{T\left(T_{m}^{0}-T\right)}\right],$$

(2) $$T_{\infty }=T_{g}-30\;{}^{\circ}C,$$

(3) $$f=\frac{\left(T+T_{m}^{0}\right)}{2T},$$

where $G_{0}$ and $K_{g}$ are material grade-specific constants which can be determined under quiescent conditions, $U^{*}$ is the activation energy of motion, $R_{g}$ is the gas constant, $T_{g}$ is the glass transition temperature, $T_{m}^{0}$ is the material grade-specific equilibrium melting temperature which is assumed to depend on pressure only, and $T_{\infty }$ is a measure of the thermal stability of the material. A linear function is chosen to describe the pressure dependence:

(4) $$T_{m}^{0}=T_{eql}+b_{6}P,$$

where $T_{eql}$ is the equilibrium melting temperature, $b_{6}$ is a grade-specific constant of the PVT model for the material, and P is the pressure [30].

The total number of small nuclei from which the structures growth proceed is given by the sum of the number of activated nuclei in the quiescent condition, $N_{0}$, and the number of activated nuclei induced by the flow, $N_{f}$:

(5) $$N=N_{0}+N_{f}.$$

The number of activated nuclei under the quiescent condition $N_{0}$ was investigated in the past [27,31] and is assumed to be a unique function of the supercooling temperature ∆T according to

(6) $$lnN_{0}=a_{N}\Delta T+b_{N}={a_{N}(T}_{m}^{0}-T)+b_{N}.$$

where $a_{N}$ and $b_{N}$ are material grade-specific constants [22,25].

The number of active nuclei induced by the flow is given by a linear first order differential equation [32] in which the RHS (known term) is dependent upon flow. Therefore, the RHS is considered dependent upon temperature, excess of free energy under quiescent conditions, $\Delta F_{q}$, and the excess of free energy under flow conditions, $\Delta F_{f}$.

(7) $$\begin{array}{c} {\dot{N}}_{f}+\frac{1}{T_{\_rlx}}N_{f}=\\ =C_{0}k_{B}T\;exp\left(-\frac{U^{*}}{R_{g}\left(T-T_{\infty }\right)}\right)\{(\Delta F_{q}+\\ {\left(\Delta F_{f}\right)}^{q\_indx})exp\left(-\frac{\Phi \left(T\right)K_{g}}{2T^{2}\left[\left(\left(1+\vartheta {\left(\Delta F_{f}\right)}^{q\_indx}\right)T_{m}^{0}-T\right)\right]}\right)-\Delta F_{q}exp(-\frac{\Phi \left(T\right)K_{g}}{2T^{2}\Delta T})\}, \end{array}$$

where $k_{B}$ is the Boltzmann constant, $C_{0}$, $T_{\_rlx}$ and q_indx are model parameters. The free Gibbs energy under quiescent conditions, $\Delta F_{q}$, the factors ϑ and $\Phi \left(T\right)$ are given by

(8) $$\Phi \left(T\right)=C_{1}\left(T+T_{m}^{0}\right),$$

(9) $$\vartheta =\frac{T_{m}^{0}}{\Delta H_{0}T},$$

(10) $$\Delta F_{q}=\Delta H_{0}\Delta T/T_{m}^{0}=\Delta H_{0}{(T}_{m}^{0}-T)/T_{m}^{0},$$

where $∆H_{0}$ is the material latent heat of crystallization [33] automatically calculated from the area of the spike exbited by the specific heat data of the semicrystalline material.

**Figure 1:**
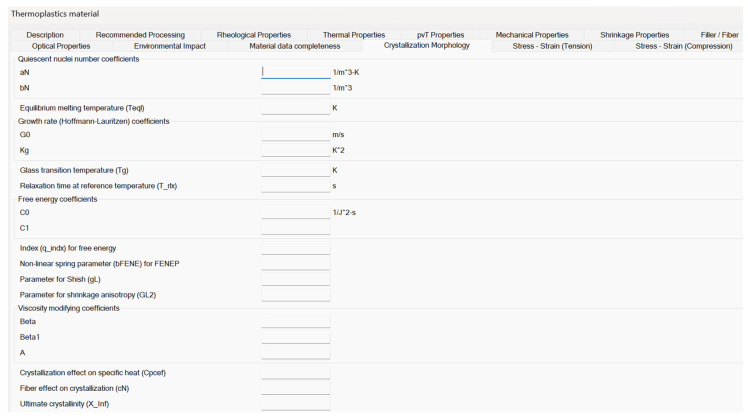
Moldflow crystallization morphology tab for thermoplastic materials. All the parameters discussed in this work must be inserted in this tab.

Figure 1 shows the crystallization morphology tab for thermoplastic materials in Moldflow. In this tab, the values of most of the parameters adopted by the models describing quiescent and flow-induced crystallization can be inserted, as well as those parameters adopted by the models describing the interplay between crystallinity and other material properties. However, some model parameters adopted in the previous equations are not directly editable in this tab because are evaluated from different material property tab (as in the case of $∆H_{0}$ and $b_{6}$) or are considered fixed (as in the case of $U^{*}$, which is assumed constant and equal to 6270 J/(mol K)), as provided by the technical support of Moldflow.

## 4. Isothermal Simulation in Moldflow

By selecting the thermoplastics injection molding process and adopting the Filling analysis for semi-crystalline material, Moldflow software performs the non-isothermal numerical simulation of the polymer mold filling, giving as output, among other things, temperature evolutions at different distances along the mold thickness direction in fixed positions along the polymer flow direction. The calculations made by Moldflow are rather complex, and so is the analysis of crystallization kinetics. The effect of the different variables on the crystallization kinetics, although briefly described in the user’s manual, is not fully clear.

**Figure 2:**
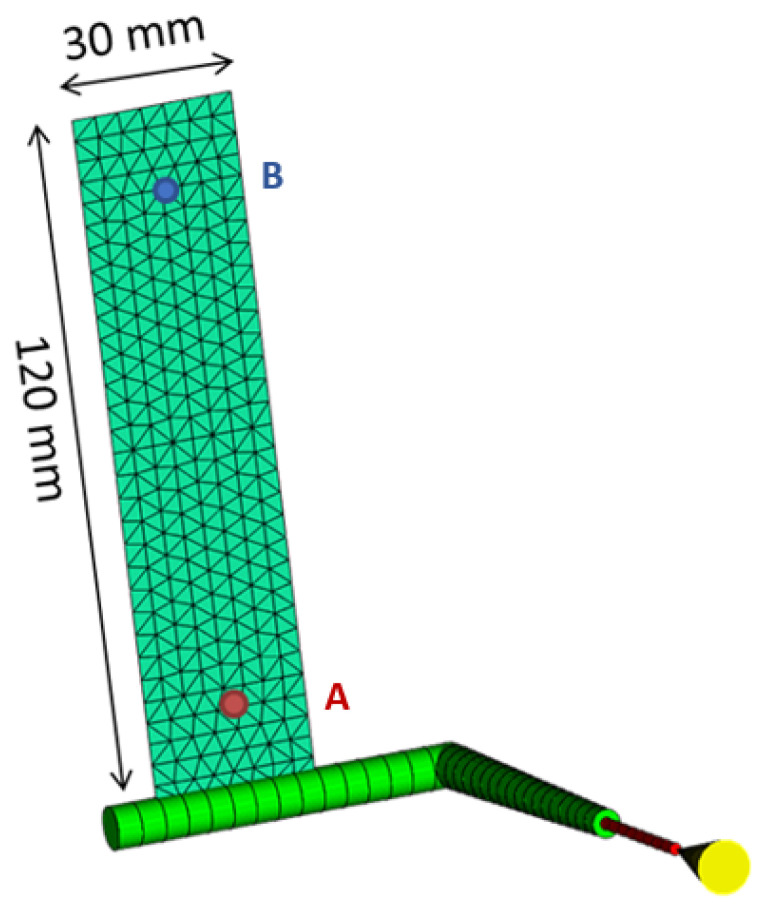
Part geometry adopted for isothermal simulations (part thickness 5 mm).

Therefore, in order to understand the crystallization kinetics implemented by Moldflow, isothermal numerical simulations of semi-crystalline polymers were implemented. To this end, filling analyses for semi-crystalline polymer were performed, adopting, in the study process conditions, the same value for mold and melt temperatures. Such a value was always selected to be larger than the polymer no-flow temperature. The geometry adopted for isothermal simulations is reported in Figure 2.

**Figure 3:**
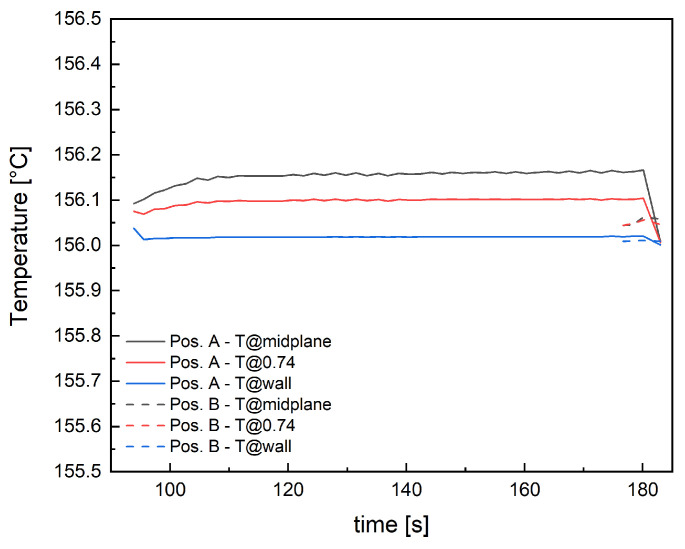
Temperature evolution for the three positions analyzed in position A and position B.

Among the simulation outputs, temperature evolution in the part thickness during the filling simulation was analyzed at two cavity positions: 10 mm from the gate (position indicated as A in Figure 2) and 10 mm from the end of the part (position indicated as B in Figure 2). Temperature profiles resulting from an isothermal simulation at 156 °C are reported in Figure 3. The thermal profiles were considered at three distances along normalized thickness: at 1, corresponding to the wall, 0.74 and at 0, corresponding to the midplane.

The filling time of the whole geometry for the isothermal simulation at 156 °C is 183 s. The material reaches position A after 94 s, remaining in the range 156–156.16 °C throughout the thickness. After 177 s, the material reaches position B, with a temperature range of 156–156.06 °C. Therefore, during the cavity filling, that occurs in 90 s, the material can be considered under isothermal conditions. This simulation will be adopted to test the isothermal crystallization of the polymer.

## 5. Injection Molding Simulation in Moldflow—Crystallization Morphology Prediction

The challenging task of making predictions from numerical simulations more reliable and precise is highly relevant and always topical. To this aim, a more complete and affordable description of the process, in terms of both processing conditions and material description, is required. For example, in the case of a semi-crystalline polymer, completing the material description by adding its crystallization kinetics, both under quiescent and flow conditions, can significantly improve the prediction accuracy. More precise predictions of both processing variable evolutions (i.e., pressure, temperature, velocity) and morphological distribution that affect the mechanical properties lead to more reliable shrinkage and warpage predictions. However, this prediction improvement can only be achieved by defining the relevant crystallization data, specifically the crystallization morphology properties of the semicrystalline polymer. The determination of these properties, according to the crystallization kinetics adopted by Moldflow, is a time-consuming procedure with a non-negligible cost. However, this issue can be overcome when pre-existent polymer crystallization kinetics are available, even if the crystallization description adopted does not adhere to the Moldflow crystallization model. In the following parts of this study, we will show how to correctly implement in Moldflow two different quiescent crystallization kinetics already defined in the literature [25,34].

### 5.1. Crystallization Kinetics Adopting Spherulitic Heterogeneous Nucleation

Crystallization process of the iPP considered in this work was experimentally investigated [12]. The crystallinity evolution was described by a Kolmogoroff–Avrami model [12] (named UNISA in the following) which accounts for the evolution of isotropic structures (spherulites) coupling a nucleation and a growth process. Spherulitic heterogeneous nucleation was identified under quiescent conditions for iPP considered in this work. In the hypothesis of heterogeneous nucleation, spherulitic nucleation density as a function of temperature was described by Equation (11):

(11) $$N\left(T\left(t\right)\right)=\frac{N_{0}}{1+A_{n}exp\left(B_{n}\left(T-T_{m,0}\right)\right)},$$

where $N_{0}$, $A_{n}$, $B_{n}$ are parameters that describe the dependence of the spherulitic nucleation density with the temperature, $T_{m,0}$ is the equilibrium melting temperature, above which crystallization occurs. Values of the parameters of Equation (11) are shown in Table 1.

**Figure 4:**
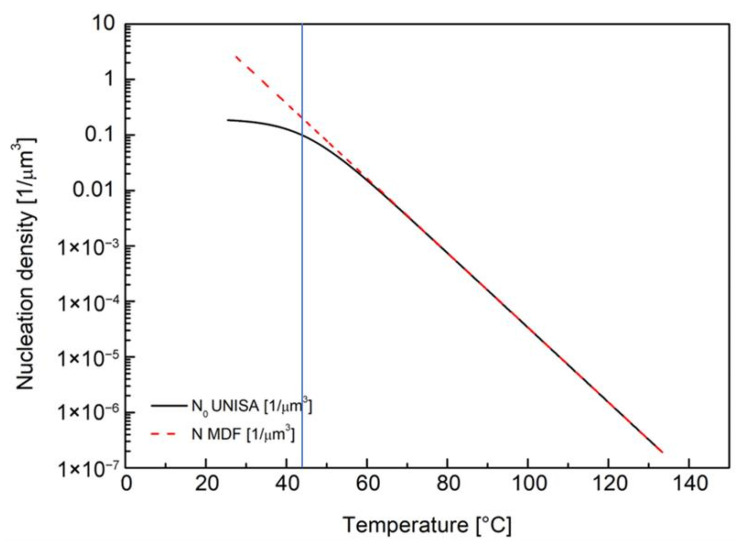
Comparison between nucleation density N as calculated for T30G UNISA experimental model (solid black line) and for Moldflow (dotted red line). The vertical line identifies the temperature at which the values predicted by the two equations differ by a factor two.

For the iPP considered in this work, the nucleation density increases as the temperature decreases, eventually reaching a plateau equal to $N_{0}$ for high undercooling. It is clear that there is no possibility of describing such an effect by using the equation implemented in Moldflow (Equation (6)) since that equation does not consider a plateau at low temperatures. The best possible match between Equation (6) (the one implemented in Moldflow) and Equation (11) (the one describing the real behavior of heterogeneous nucleation of the iPP considered in this work) is to choose the parameters of Equation (6) in such a way as to describe the exponential dependence of the nucleation density on the temperature at high temperatures, as shown in Figure 4. The parameters used in Equation (6) for iPP T30G are reported in Table 2.

Below 60 °C, the temperature dependence of nucleation described by the Moldflow equation (Equation (6)) predicts a larger number of nuclei, as shown in Figure 4. The nucleation density predicted by Moldflow is larger by a factor of two than the one describing the real behavior of the material when the temperature is equal to 42 °C.

In the UNISA model, the growth rate of the iPP considered in this work was described by the Hoffman and Lauritzen expression, Equation (1). However, differently from the Moldflow model, the iPP crystallization kinetics was defined considering $U^{*}$, the activation energy of motion in the growth rate dependence upon temperature, a material grade-specific constant. Values of the parameters adopted for iPP spherulitic growth rate are shown in Table 3.

In order to perform numerical simulation with the iPP selected in this work, the parameters in the Crystallization Morphology tab (shown in Figure 1) have to be set. However, due to the differences between the iPP nucleation density model experimentally developed at UNISA and the one implemented in Moldflow, parameters shown in Table 1 and Table 3 cannot be directly introduced as crystallization data. It was decided to compare the two models by considering half crystallization time $t_{0.5}$. Equation (12) defines $t_{0.5}$ at temperature T under isothermal conditions.

(12) $$t_{0.5}(T)=\frac{1}{G(T)}\sqrt[{3}]{\frac{3\ln2}{4\pi N(T)}},$$

The parameters to be adopted in Moldflow crystallization morphology tab were obtained by matching the isothermal half crystallization times calculated according to Equation (12) using the two different models. iPP isothermal half crystallization times were calculated in an Excel worksheet for UNISA crystallization model by substituting Equation (11) and Equation (1) into Equation (12) and then using parameters values as reported in Table 1 and Table 3, respectively. The parameters for the Moldflow model were determined using the generalized reduced gradient (GRG) model of Excel Solver, minimizing the absolute deviation between the two evaluations of isothermal half crystallization times. The parameters obtained from the half crystallization times comparison are shown in Table 2.

**Figure 5:**
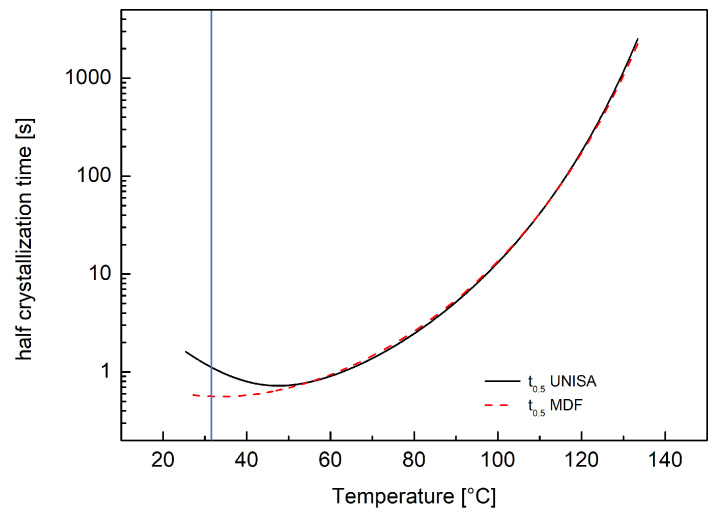
Comparison between half crystallization time calculated with N and G for T30G UNISA experimental model (solid black line) and N and G calculated according to Moldflow equations (dotted red line). The vertical line identifies the temperature at which the values predicted by the two models differ by a factor two.

Figure 5 compares half crystallization times evaluated with UNISA and Moldflow models. A perfect agreement between the two models can be observed at high temperatures, where the two models for nucleation density coincide. Some discrepancies are found at temperatures lower than 50 °C. However, a factor of two in the predictions obtained by the two models is found at a temperature of 31 °C. This means that a lower growth rate implemented in Moldflow compensates for the larger nucleation rate in the range from 42 °C to 31 °C. Due to the fast crystallization times at temperatures close to 50 °C (about 1 s) it can be stated that below 50 °C the polymer is probably already completely crystallized and thus the discrepancy at low temperatures is of minor importance.

### 5.2. Simulations with Spherulitic Heterogeneous Nucleation

**Figure 6:**
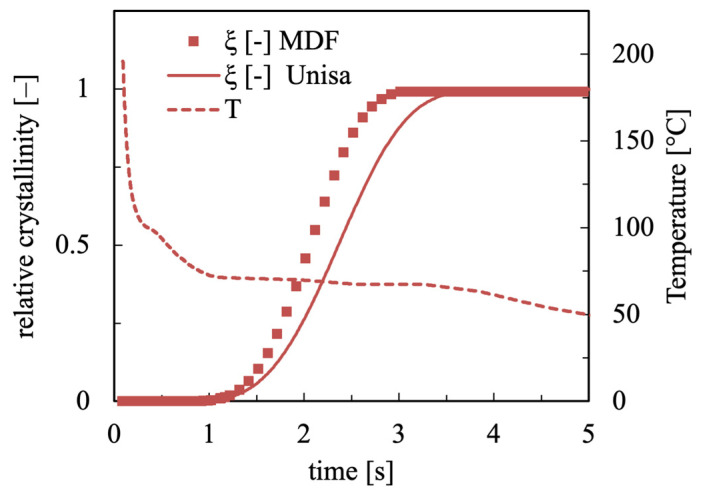
Crystallinity evolution as obtained by Moldflow simulation and by UNISA experimental model simulation for samples obtained at 25 °C mold temperature. For the same test, temperature evolution calculated with Moldflow is shown. Crystallinity and temperature are calculated at 10 mm from the gate and 0.86 normalized distance (0 corresponds to midplane and 1 to cavity wall) from the sample midplane.

Moldflow simulations were conducted for iPP, adopting a rectangular cavity and the injection conditions described in the Materials and Methods section, along with a 25 °C mold temperature, and giving the crystallinity evolution along sample thickness (0 corresponds to midplane and 1 to cavity wall) for an investigated position at 10 mm from the gate as an output. The iPP UNISA crystallization model was implemented in an Excel worksheet. Using the evolution of temperatures obtained from Moldflow simulations as input, the crystallinity evolutions at each position along the sample thickness were calculated and then compared with Moldflow crystallinity outputs. In Figure 6, the comparison between crystallinity evolution predicted by Moldflow (MDF) and from the experimental model (UNISA) was shown at 0.86 normalized distance from midplane. Also, the temperature evolution calculated with Moldflow is shown for the duration of the crystallization process.

The material solidifies in a few seconds, at temperatures larger than 50 °C, where the two models essentially coincide. However, Moldflow simulations anticipate crystallization, with the complete crystallization obtained almost one second before the UNISA model. This is probably due to the effect of pressure on the crystallization kinetics described by Moldflow with Equation (4) which cannot be changed.

**Figure 7:**
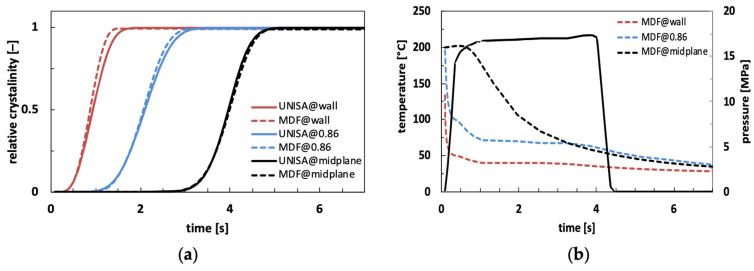
Crystallinity evolution as obtained by Moldflow simulation (MDF) and by experimental model (UNISA) for samples obtained at 25 °C mold temperature at 10 mm from the gate for three positions along thickness: wall, 0.86 and midplane (**a**); temperature evolution for the three positions analyzed and pressure profile at injection point calculated by Moldflow (**b**).

Also, considering the effect of pressure in the UNISA crystallization model, Figure 7a shows the comparison between crystallinity evolution as obtained from Moldflow and from the UNISA model for three positions along sample thickness. Figure 7b shows temperature profiles for the three positions considered and pressure profile.

Predictions with the UNISA model were carried out considering a linear dependence of $T_{m,0}\;$ on pressure according to Equation (4). In accordance with Moldflow simulation, a value of 6.25 × 10^8^ K/Pa was adopted for $b_{6}$. At the cavity wall, the polymer instantly crystallizes, while pressure is still increasing, so the effect of pressure is negligible. Material crystallization occurs at low temperatures, where two models exhibit some discrepancies in calculated half crystallization time as shown by Figure 5 and thus the agreement between their predictions is not perfect. At the midplane, as crystallization times are longer, the pressure profile is fully developed during the crystallization process. In this position, predictions of both models collapse, confirming the consistency of the two models. At intermediate position (i.e., 0.86), differences between the two predictions are reduced with respect to the previous comparison shown in Figure 6. However, some discrepancies between the two predictions remain, which can be ascribed to the pressure increase occurring in the first second of the simulation.

**Figure 8:**
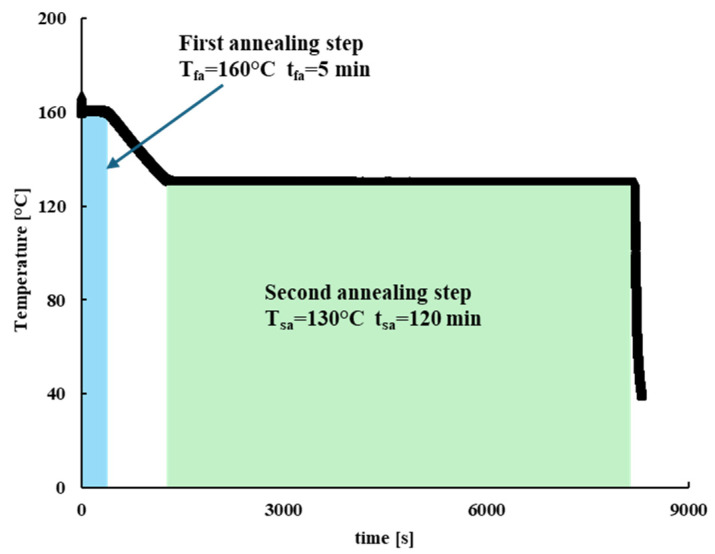
The black line represents the thermal protocol adopted for the two-step annealing injection molding run.

To further validate the crystallinity Moldflow simulations, a more complex injection molding test was conducted with a HAAKE Minijet II with a non-constant mold wall temperature. In particular, a two-step in-mold annealing protocol was adopted. A first step of 160 °C mold temperature during the filling and packing stages, followed by 5 min annealing time, was performed. After that, a second annealing step was performed at 130 °C mold temperature for 2 h, maintaining the material inside the mold. After each run the mold was quickly cooled down at 25 °C. The adopted protocol is shown in Figure 8.

**Figure 9:**
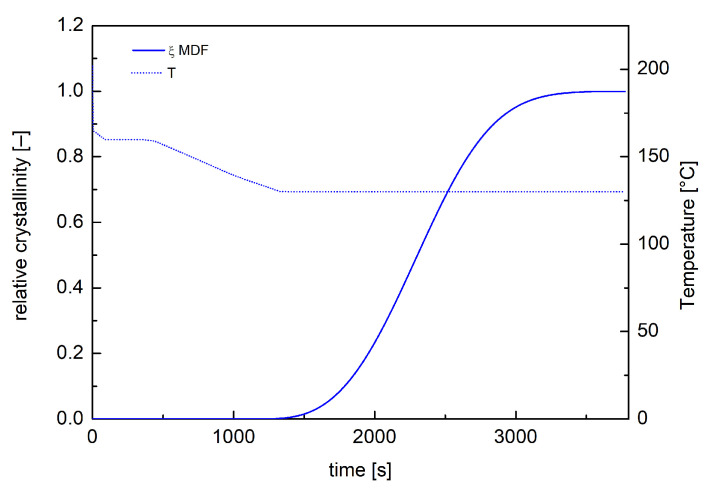
Temperature and crystallinity evolutions calculated by Moldflow simulations, at 10 mm from the rectangular cavity entrance at a 0.06 normalized distance from the midplane, for the sample obtained by two-step annealing protocol.

Moldflow simulations were conducted adopting a dual-domain mesh composed of 12,354 triangular elements. Moldflow crystallization analyses of the whole process were carried out by modifying the Part Surface mold temperature and by defining, in the Mold Temperature Profile tab, a temperature profile reproducing the thermal protocol shown in Figure 8. Moldflow outputs were generated at 10 mm from the rectangular cavity entrance at different positions along the sample half-thickness. Figure 9 shows temperature and crystallization profile close to the sample mid-plane during the two-step annealing test as obtained from Moldflow simulation. In this case, crystallization occurred during the isothermal step at 130 °C, reaching the maximum allowable crystallinity degree.

This behavior occurs for most of the normalized distance resulting in an essentially uniform distribution of the predicted overall crystallinity along the sample thickness. This is consistent with previous experimental findings [35], which showed an almost constant distribution of the overall crystallinity degree along the thickness of the iPP molded sample.

**Figure 10:**
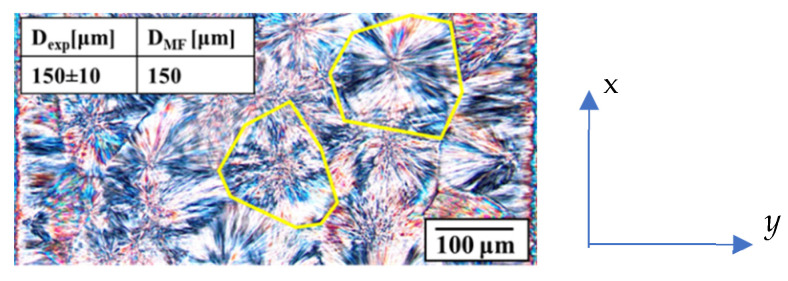
Optical image of spherulites inside the sample obtained with two-step annealing protocol reported in Figure 8. *x* = flow direction, *y* = thickness direction. The yellow lines indicate examples of spherulite boundaries. Comparison between experimental spherulitic average diameter and as obtained from Moldflow simulations is reported in the insight.

Optical micrographs of slices cut at 10 mm from the rectangular cavity entrance allowed the morphological observations of the whole sample thickness. In particular, along the sample thickness, the presence of a reduced fibrillar area close to the sample wall was evidenced with a large area characterized by isotropic structures (spherulites). Analyses of optical micrographs with an image processing software (ImageJ) allowed the measurement of the spherulitic diameter within sample thickness. In particular, to determine the average value of the dimension of the spherulites, slices from three different samples were cut at 10 mm downstream of the rectangular cavity entrance in the *x–y* plane (namely the flow-thickness plane), as shown in Figure 10. Each slice had dimensions of 1 mm in the y-direction (corresponding to the whole sample thickness) and 0.8 mm in the flow direction. In the resulting micrograph, each spherulite was highlighted in order to evaluate the area. Figure 10 shows representative examples of highlighted structures. Assuming an equivalent circle, the diameter of all spherulites detectable within the observed area was calculated. The diameter distribution along the sample thickness was essentially flat.

Figure 10 shows an optical image of the spherulitic layer within the sample obtained with the two-step annealing protocol. Figure 10 shows only a part of the slice used to measure the spherulites diameters in the sample, specifically the central area close to the sample mid-plane. The spherulites diameter values experimentally determined from slice image analysis, $D_{exp}$, and the predicted ones from Moldflow simulations, $D_{MF}$, are reported in the inset of Figure 10. Predicted diameter distribution is flat, and values are almost identical.

In particular, in Figure 9, simulations reveal that the formation of a reduced number of nuclei occurred during the second annealing step at 130 °C and they had time to grow. In these conditions, crystallization occurred under isothermal and quiescent conditions, in other words, under conditions like those adopted during the experimental investigation of iPP crystallization phenomenon. This means that the quiescent model UNISA implemented in the Moldflow crystallinity analysis is accurate in describing crystallization and, consequently, in predicting the final dimensions of the morphological features.

### 5.3. Crystallization Kinetics Adopting Spherulitic Homogeneous Nucleation

Previous experimental research on the quiescent crystallization behavior of polyoxymethylene (POM) revealed a nucleation phenomenon that is the result of a heterogeneous nucleation process, which increases with decreasing temperature, and a prevailing homogeneous nucleation process, represented by an increase in crystalline structures with time [25]. The crystallization kinetics were described by a Kolmogoroff–Avrami model (UNISA-POM in the following) as already done for iPP, which considers the coupling of nucleation and growth processes. The dependence of growth rate upon temperature is expressed by Equation (1). Unlike the iPP, the nucleation can be expressed as the sum of the number of predetermined nuclei in the quiescent condition $N_{0}\left(T\right)$ (Equation (6)) and a term representing the homogeneous nucleation $N_{h}\left(T\right)$, that can be evaluated by the Hoffman–Lauritzen theory:

(13) $$N=N_{0}\left(T\right)+N_{h}\left(T\right),$$

(14) $${\dot{N}}_{h}(T){=D}_{1}exp\left[-\frac{D_{2}\left(T+T_{m}\right)}{2T^{2}\left(T_{m}-T\right)}\right],$$

Values of parameters adopted in Equation (14) to describe the dependence upon temperature of the rate of homogenous nucleation are reported in Table 4.

The overall nucleation process description is similar for the UNISA-POM model (Equation (13)) and Moldflow model (Equation (5)). The main difference concerns the rate of the nucleation term added to the heterogeneous nucleation used by UNISA-POM model (Equation (14)) and Moldflow model (Equation (7)). In particular, the form of the differential equation and the expression of the right-hand side, RHS, (which is considered dependent upon flow and temperature in the Moldflow model) are different. Therefore, to simulate the quiescent crystallization behavior of POM on Moldflow, we first adopted a high relaxation time that makes the zero-order differential term in the left-hand side, LHS, negligible. Secondly, by considering the parameter *q__indx_* to zero, the dependence of flow in the RHS was eliminated.

**Figure 11:**
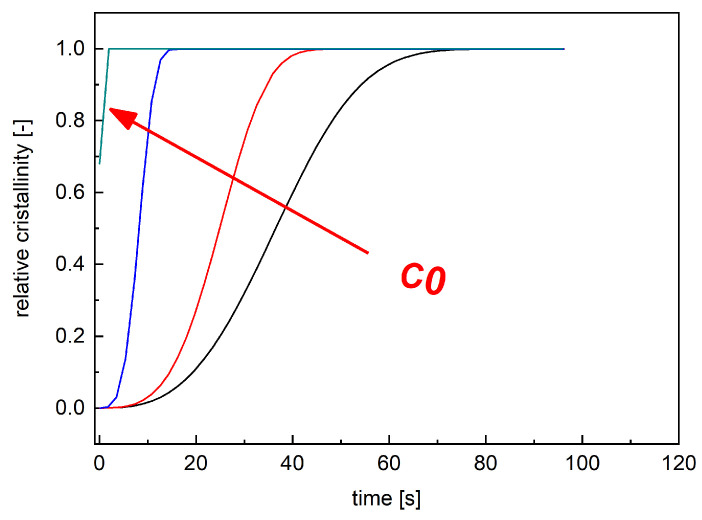
Lines with different colors represent predicted crystallinity evolution under isothermal conditions at 154 °C obtained with different values of $C_{0}$. Heterogeneous nucleation is set to zero in these simulations.

By considering these assumptions, isothermal simulations at 154 °C were carried out adopting Moldflow. Isothermal simulations were conducted assuming $C_{1}=0.1$ with different values of $C_{0}$ by adopting the geometry shown in Figure 2. Figure 11 shows the crystallinity evolution in position A, illustrated in Figure 2 for isothermal simulations obtained with different values of $C_{0}$.

By imposing $C_{0}=0$, the variation in crystallization degree is slower since the nucleation process is only due to the heterogeneous term. By increasing the values of $C_{0}$, the crystallization degree increases faster due to the extra homogeneous nuclei generation (Figure 11). To complete the morphological crystallization data, crystallinity evolutions under different isothermal conditions predicted by Moldflow were compared with the predictions of the UNISA-POM model. Such predictions were carried out with an Excel ad hoc developed procedure implementing the crystallinity evolution model of UNISA-POM that adopts Moldflow temperature evolution as input. By comparing the results of the two models’ simulations, a value of $C_{0}=1\;\times \;10^{-29}$ was chosen.

The parameters adopted in the Moldflow crystallization morphology tab to simulate the quiescent crystallization behavior of POM are reported in Table 5.

**Figure 12:**
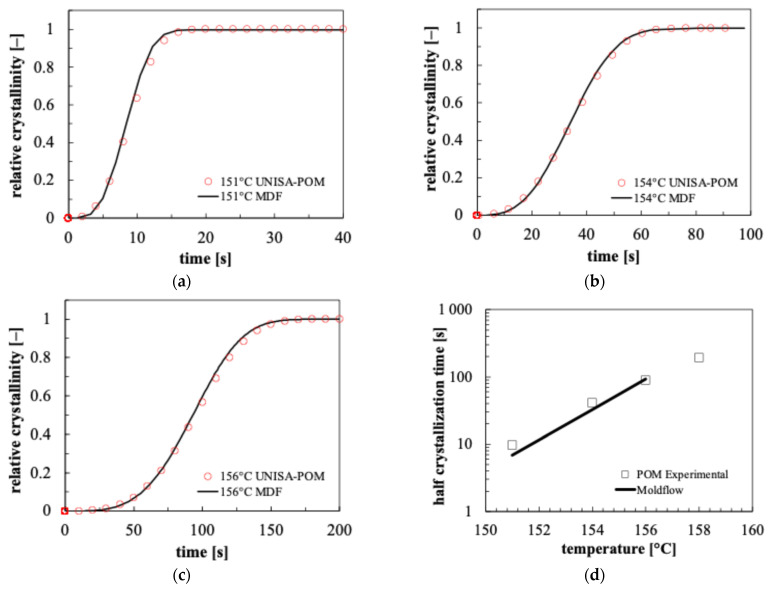
Comparison between crystallinity evolutions calculated by Moldflow adopting the parameters of Table 4 (solid line) and by the UNISA-POM model simulations (symbols) at different isothermal temperatures: 151 °C (**a**); 154 °C (**b**); 156 °C (**c**). Comparison between experimental half crystallization times and values calculated with Moldflow isothermal simulations (**d**).

Figure 12 shows the comparison of different isothermal crystallinity evolutions obtained by Moldflow simulations conducted with the parameters reported in Table 4 and by Excel simulations conducted with the UNISA-POM model. Furthermore, a comparison between POM experimental half crystallization time and the values predicted by isothermal Moldflow simulations is also reported in Figure 12.

Calculated crystallinity evolutions by Moldflow simulations and UNISA-POM model simulations compare favorably at each isothermal temperature (Figure 12a–c). Moldflow’s predicted half crystallization times were compared with POM experimental values measured by calorimetric tests [25]. Isothermal Moldflow simulations accurately predict calorimetric half crystallization times.

**Figure 13:**
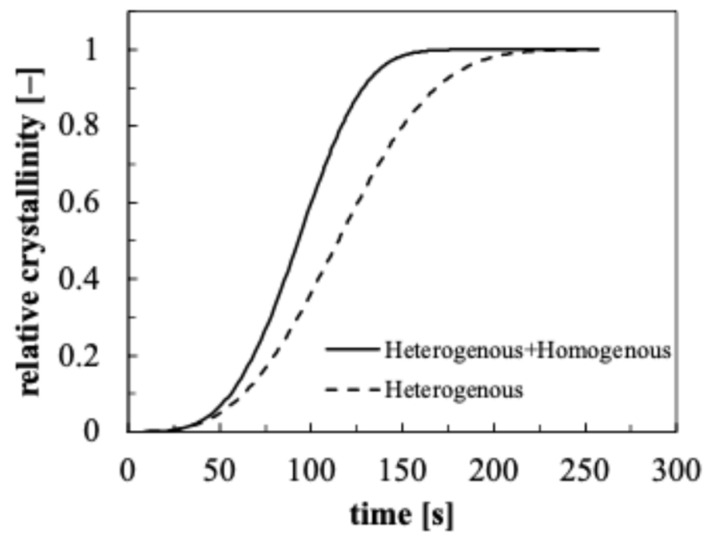
Crystallinity evolution calculated by Moldflow simulations at 156 °C in two conditions: nucleation that takes into account of both heterogenous and homogenous mechanisms (solid line), and nucleation that considers only the heterogenous mechanism (dashed line).

To evaluate the relevance of homogenous nucleation on POM crystallization kinetics, isothermal Moldflow simulations were carried out. Figure 13 shows the crystallinity evolution calculated by Moldflow isothermal simulations at 156 °C in two conditions: nucleation that takes into account of both heterogenous and homogenous mechanisms, and nucleation that considers only the heterogenous mechanism.

As expected, at this temperature, neglecting the homogenous nucleation mechanism reduces the overall crystallization rate, which leads to POM complete crystallization over a longer time.

**Figure 14:**
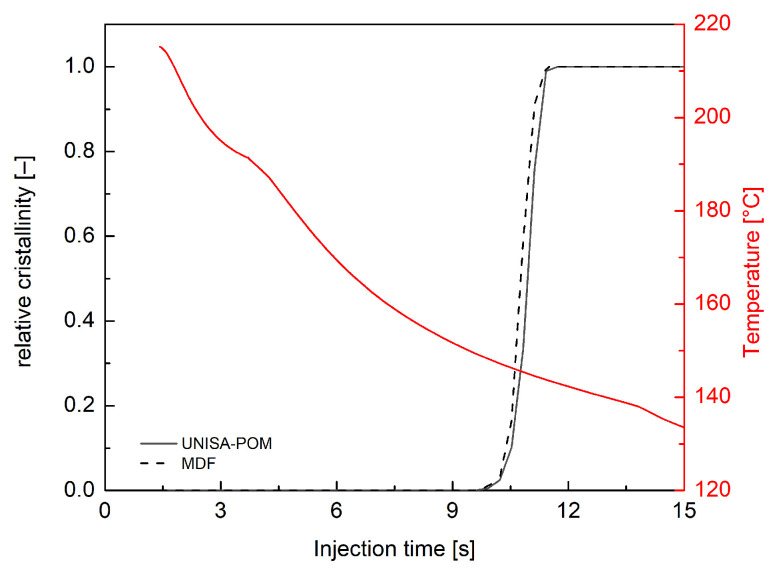
Temperature and crystallinity evolutions calculated by Moldflow simulations at 0.55 mm from the wall (solid lines) and crystallinity predictions from UNISA-POM model (dotted line).

To further validate the parameters of the crystallization morphology tab Moldflow defined, non-isothermal simulations were performed by Moldflow. According to the default process conditions indicated by the Moldflow database, a 215 °C melt temperature and a 90 °C mold temperature were used. The calculated temperature and the crystallinity evolution in position A at 0.55 mm from the wall (solid lines) are shown in Figure 14. The crystallinity prediction obtained with Excel simulations of the UNISA-POM model is shown (dotted line).

Concerning the Moldflow simulation, the temperature decreases from a value close to the injection temperature to the 90 °C mold temperature. The crystallization degree starts only when the temperature drops down to 160 °C. The plot shows a good agreement between the crystallinity evolution resulting from Moldflow simulations conducted with the parameters shown in Table 4 and the results of the Excel simulation implementing the UNISA-POM model. Convergence between the prediction of the two models is also confirmed in a realistic injection molding test.

## 6. Conclusions

Moldflow numerical simulations allow us to obtain reliable predictions of process-variable evolutions, that in turn determine the final properties of the molded parts, only if the software adopts an accurate and complete description of the material behavior. For semi-crystalline materials, the injection molding simulations have to be performed by considering the crystallization analysis to improve the predictions accuracy. That implies the creation of the material crystallization database with the parameters of the Moldflow crystallization kinetics model (based on the Avrami–Kolmogoroff kinetics model). However, this generation is not a straightforward process due to the differences frequently occurring between the kinetics models implemented experimentally and in Moldflow software. In this work, the approaches followed to correctly generate a Moldflow material crystallization database of two different commercial semi-crystalline polymers are illustrated. The polymers (an iPP and a POM) have been completely characterized with respect to the quiescent crystallization phenomena, adopting kinetics models different from the Moldflow crystallization model. Both isothermal and non-isothermal Moldflow simulations of a rectangular cavity have been considered to show the convergence between the Moldflow model and the polymer experimental model. Unlike the Moldflow crystallization model, experimental iPP crystallization kinetics assumes that nucleation density exhibits a constant value for high levels of undercooling. The material database created assures a perfect agreement between the half crystallization time at high temperatures, while below 50 °C lower crystallization times are predicted by Moldflow. However, due to the fast crystallization in this range of temperatures, the discrepancies between the two models are not relevant. Non-isothermal simulations were conducted showing that Moldflow correctly reproduced the iPP experimental behavior. In particular, at several distances from the sample mid-plane, by considering the same pressure effect on crystallization for the two models, crystallinity evolution predictions obtained by the two models collapse, exhibiting minor discrepancies at distances where crystallization occurs at low temperature. Moldflow crystallization analysis was also validated in a more complex iPP experimental injection test. In particular, the test was conducted with a non-constant mold wall temperature. Moldflow simulations were carried out by adopting the registered mold wall distribution. Moldflow crystallization analysis calculated predicted spherulites diameters almost identical to the experimentally observed values. Moreover, a POM that experimentally exhibits a nucleation phenomenon, resulting in a prevailing homogeneous process with respect to heterogenous process, was considered. Moldflow database parameters were determined to describe the overall nucleation process. Isothermal crystallinity evolutions calculated by Moldflow simulations and by experimental models compare favorably. Furthermore, half crystallization times calculated by isothermal Moldflow simulations accurately predicted the measured calorimetric ones. Finally, non-isothermal simulation confirmed the ability of Moldflow to describe the POM experimental model in a realistic injection molding test.

## Figures and Tables

**Table 1:** Values of parameters adopted by the UNISA model in Equation (11) to describe the temperature dependence of the spherulitic nucleation density for the iPP considered in this work.

Parameter	Value
$N_{0}$ [1/m^3^]	1.95 × 10^5^
$A_{n}$ [–]	1.3 × 10^8^
$B_{n}$ [K^−1^]	0.155
$T_{m,0}$ [K]	467.54

**Table 2:** Moldflow crystallization morphology data adopted in simulations with iPP T30G. These parameters have to be inserted in the tab reported in Figure 1.

Parameter	Value
$a_{N}$ [1/m^3^K]	0.155
$b_{N}$ [1/m^3^]	16.5235
$T_{eql}$ [K]	467.54
$G_{0}$ [m/s]	7.716
$K_{g}$ [K^2^]	356,113
$T_{g}$ [K]	225.3

**Table 3:** Values of parameters adopted by UNISA model in Equation (1) to describe the temperature dependence of the spherulitic growth rate for the iPP considered in this work.

Parameter	Value
$U^{*}/R_{g}$ [K]	751.6
$G_{0}$ [m/s]	13.80
$K_{g}$ [K^2^]	371,381
$T_{m,0}$ [K]	467.54
$T_{\infty }$ [K]	198.46

**Table 4:** Values of parameters adopted by UNISA-POM model in Equation (14) to describe the temperature dependence of the homogeneous nucleation rate for the POM considered in this work.

Parameter	Value
$D_{1}$ [1/(μm^3^ s)]	6.06 × 10^2^
$D_{2}$ [K^2^]	2.88 × 10^5^
$T_{m,0}$ [K]	465

**Table 5:** Value of parameters that describe the quiescent crystallization behavior of POM in Moldflow.

Parameter	Value
$a_{N}$ [1/m^3^K]	0.75
$b_{N}$ [1/m^3^]	4.3284
$T_{eql}$ [K]	462.5
$G_{0}$ [m/s]	7.05
$K_{g}$ [K^2^]	225,270
$T_{g}$ [K]	200
$T_{\_rlx}$ [s]	9 × 10^11^
$C_{0}$ [1/J^2^s]	1 × 10^−29^
$C_{1}$ [–]	0.1
q_indx [–]	0
bFENE [–]	0
X_inf [–]	0.6

## Data Availability

The data that support the findings of this study are available from the corresponding author upon reasonable request.

## References

[1] Chiang H.H., Hieber C.A., Wang K.K. (1991). A Unified Simulation of the Filling and Postfilling Stages in Injection Molding. Part I: Formulation. Polym. Eng. Sci..

[2] Pantani R., Speranza V., Titomanlio G. (2016). Thirty Years of Modeling of Injection Molding. A Brief Review of the Contribution of UNISA Code to the Field. Int. Polym. Process..

[3] Li X., Wei Q., Li J., Yang J., Guan J., Qiu B., Xu J., Wang X. (2019). Numerical Simulation on Crystallization-Induced Warpage of Injection-Molded PP/EPDM Part. J. Polym. Res..

[4] Zhang J., Yu K., Luo Y., Li W., Zhong X., Liu G., Bao J., Chen C. (2025). Isothermal Crystallization Kinetics and Their Effect on the Molding Process and Mechanical Properties of PAEK and PEEK. Polymers.

[5] Estrella-Guayasamin M., Figueroa-López U., Guevara-Morales A., García-León R.A. (2023). Effect of Crystallization and Packing Pressure on the Development of Residual Stresses on Injection Molded Polypropylene Samples. Polym. Bull..

[6] Saad S., Cruz C., Régnier G., Ammar A. (2024). Efficient Identification of a Flow-Induced Crystallization Model for Injection Molding Simulation. Int. J. Adv. Manuf. Technol..

[7] Avrami M. (1939). Kinetics of Phase Change. I General Theory. J. Chem. Phys..

[8] Ozawa T. (1971). Kinetics of Non-Isothermal Crystallization. Polymer.

[9] Nakamura K., Watanabe T., Katayama K., Amano T. (1972). Some Aspects of Nonisothermal Crystallization of Polymers. I. Relationship between Crystallization Temperature, Crystallinity, and Cooling Conditions. J. Appl. Polym. Sci..

[10] Kim S.K., Jeong A. (2019). Numerical Simulation of Crystal Growth in Injection Molded Thermoplastics Based on Monte Carlo Method with Shear Rate Tracking. Int. J. Precis. Eng. Manuf..

[11] Nie C., Peng F., Cao R., Cui K., Sheng J., Chen W., Li L. (2022). Recent Progress in Flow-induced Polymer Crystallization. J. Polym. Sci..

[12] Speranza V., Liparoti S., Volpe V., Titomanlio G., Pantani R. (2020). Modelling of Morphology Development towards Spherulites and Shish–Kebabs: Application to Isothermal Flow-Induced Crystallization Experiments on Isotactic Polypropylene. Polymer.

[13] Vázquez L.S., Pereira M., Díaz-Díaz A.-M., López-Beceiro J., Artiaga R. (2024). Isothermal Crystallization Kinetics of Commercial PA66 and PA11. J. Therm. Anal. Calorim..

[14] Van Drongelen M., Van Erp T.B., Peters G.W.M. (2012). Quantification of Non-Isothermal, Multi-Phase Crystallization of Isotactic Polypropylene: The Influence of Cooling Rate and Pressure. Polymer.

[15] Tseng C.H., Tsai P.S. (2022). The Isothermal and Nonisothermal Crystallization Kinetics and Morphology of Solvent-Precipitated Nylon 66. Polymers.

[16] López-Beceiro J., Díaz-Díaz A.-M., Fernández-Pérez E., Ferreira I., Focke W.W., Artiaga R., López-Beceiro J., Díaz-Díaz A.-M., Fernández-Pérez E., Ferreira I. (2023). A Relatively Simple Look at the Rather Complex Crystallization Kinetics of PLLA. Polymers.

[17] Mishra S., Mohanty S., Nayak S.K. (2023). Study of Nonisothermal Crystallization Kinetics of Unstretched and Uniaxially Stretched Electroactive PVDF Composite Films. Macromol. Chem. Phys..

[18] Kech A., Ludwig H.-C., Möginger B., Eyerer P., deClaville Christiansen J. (2022). Mechanical Properties of Isotactic Polypropylene with Oriented and Cross-Hatched Lamellae Structure. Int. Polym. Process..

[19] Mahmood N., Kolesov I., Glüge R., Altenbach H., Androsch R., Beiner M. (2020). Influence of Structure Gradients in Injection Moldings of Isotactic Polypropylene on Their Mechanical Properties. Polymer.

[20] Gahleitner M., Wolfschwenger J., Bachner C., Bernreitner K., Neißl W. (1996). Crystallinity and Mechanical Properties of PP-Homopolymers as Influenced by Molecular Structure and Nucleation. J. Appl. Polym. Sci..

[21] Krebelj K., Krebelj A., Halilovič M., Mole N. (2020). Modeling Injection Molding of High-Density Polyethylene with Crystallization in Open-Source Software. Polymers.

[22] Grosso G., Troisi E.M., Jaensson N.O., Peters G.W.M., Anderson P.D. (2019). Modelling Flow Induced Crystallization of IPP: Multiple Crystal Phases and Morphologies. Polymer.

[23] Coccorullo I., Pantani R., Titomanlio G. (2003). Crystallization Kinetics and Solidified Structure in IPP under High Cooling Rates. Polymer.

[24] Pantani R., Speranza V., Titomanlio G. (2015). Simultaneous Morphological and Rheological Measurements on Polypropylene: Effect of Crystallinity on Viscoelastic Parameters. J. Rheol..

[25] Volpe V., Speranza V., Schrank T., Berer M., Pantani R. (2022). An Investigation of Crystallization Kinetics of Polyoxymethylene in Processing Conditions. Polym. Adv. Technol..

[26] Zheng R., Kennedy P., Tanner R. (2004). Apparatus and Methods for Predicting Properties of Processed Material United States Patent Application.

[27] Koscher E., Fulchiron R. (2002). Influence of Shear on Polypropylene Crystallization: Morphology Development and Kinetics. Polymer.

[28] Hoffman J.D., Miller R.L. (1997). Kinetic of Crystallization from the Melt and Chain Folding in Polyethylene Fractions Revisited: Theory and Experiment. Polymer.

[29] John I., Lauritzen J., Hoffman J.D. (1960). Theory of Formation of Polymer Crystals with Folded Chains in Dilute Solution. J. Res. Natl. Bur. Stand. A Phys. Chem..

[30] Fulchiron R., Koscher E., Poutot G., Delaunay D., Régnier G. (2001). Analysis of the Pressure Effect on the Crystallization Kinetics of Polypropylene: Dilatometric Measurements and Thermal Gradient Modeling. J. Macromol. Sci. Part B.

[31] Angelloz C., Fulchiron R., Douillard A., Chabert B., Fillit R., Vautrin A., David L. (2000). Crystallization of Isotactic Polypropylene under High Pressure (γ Phase). Macromolecules.

[32] Eder G., Janeschitz-Kriegl H., Meijer H.E.H. (1997). Crystallization. Processing of Polymers.

[33] Zheng R., Kennedy P.K. (2004). A Model for Post-Flow Induced Crystallization: General Equations and Predictions. J. Rheol..

[34] Salomone R., Speranza V., Liparoti S., Titomanlio G., Pantani R. (2022). Modeling and Analysis of Morphology of Injection Molding Polypropylene Parts Induced by In-Mold Annealing. Polymers.

[35] Liparoti S., Speranza V., Titomanlio G., Pantani R. (2020). Effect of Rapid Mold Heating on the Structure and Performance of Injection-Molded Polypropylene. Polymers.

